# The strength of corticomotoneuronal drive underlies ALS split phenotypes and reflects early upper motor neuron dysfunction

**DOI:** 10.1002/brb3.2403

**Published:** 2021-10-28

**Authors:** Andrew Eisen, Peter Bede

**Affiliations:** ^1^ Division of Neurology Department of Medicine University of British Columbia British Columbia Canada; ^2^ Computational Neuroimaging Group Biomedical Sciences Institute Trinity College Dublin Dublin Ireland; ^3^ Pitié‐Salpêtrière University Hospital Sorbonne University Paris France

**Keywords:** amyotrophic lateral sclerosis, corticomuscular coherence, corticomotoneuronal, excitatory post‐synaptic potential, neuroimaging, split phenotypes

## Abstract

**Background:**

Split phenotypes, (split hand, elbow, leg, and foot), are probably unique to ALS, and are characterized by having a shared peripheral input of both affected and unaffected muscles. This implies an anatomical origin rostral to the spinal cord, primarily within the cerebral cortex. Therefore, split phenotypes are a potential marker of ALS upper motor neuron pathology. However, to date, reports documenting upper motor neuron dysfunction in split phenotypes have been limited to using transcranial magnetic stimulation and cortical threshold tracking techniques. Here, we consider several other potential methodologies that could confirm a primary upper motor neuron pathology in split phenotypes.

**Methods:**

We review the potential of: 1. measuring the compound excitatory post‐synaptic potential recorded from a single activated motor unit, 2. cortical‐muscular coherence, and 3. new advanced modalities of neuroimaging (high‐resolution imaging protocols, ultra‐high field MRI platforms [7T], and novel Non‐Gaussian diffusion models).

**Conclusions:**

We propose that muscles involved in split phenotypes are those functionally involved in the human motor repertoire used particularly in complex activities. Their anterior horn cells receive the strongest corticomotoneuronal input. This is also true of the weakest muscles that are the earliest to be affected in ALS. Descriptions of split hand in non‐ALS cases and proposals that peripheral nerve or muscle dysfunction may be causative are contentious. Only a few carefully controlled cases of each form of split phenotype, using upper motor neuron directed methodologies, are necessary to prove our postulate.

## Graphical abstract

Muscles involved in ALS split phenotypes are those with the strongest corticomotoneuronal connectivity used in complex movements developed because of evolutionary pressures.

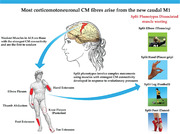

## 1 INTRODUCTION

Early upper motor neuron deficits in amyotrophic lateral sclerosis (ALS) can be elusive and difficult to identify (Swash, 2012), but they are important for early diagnosis and admission into therapeutic drug trials (Hannaford et al., 2021). Split phenotypes, in which there is dissociated muscle weakness and wasting, where some muscles are affected while others having a shared peripheral nerve and spinal nerve root innervation are spared, has been recognized for several decades as a unique feature of amyotrophic lateral sclerosis (ALS) (Wilbourn, 2000). The shared peripheral input of affected versus unaffected muscles implies the likely anatomical origin of split phenotypes in ALS is primarily within the cerebral cortex. Utility of a clinical sign depends on understanding its pathophysiology, which in turn benefits from objective methodology to accurately confirm the abnormal sign.

Despite the anatomical distribution of split phenotypes being reflective of upper motor neuron pathology, little attention has been paid to this aspect. There have been very few studies specifically using transcranial magnetic stimulation (TMS) and cortical threshold tracking, to investigate a split phenotype (Bae et al., 2014; Menon et al., 2014; Weber et al., 2000). F‐wave persistence has been used as a surrogate for upper motor neuron dysfunction in split phenotypes (Wang et al., 2019; Wang et al., 2019). This is a useful measure of spinal motoneuron excitability relating to upper motor neuron dysfunction, in particular loss of cortical inhibition, but it lacks the specificity of techniques, we discuss below, required to substantiate a cortical cause of split phenotypes.

Several split phenotypes have been reported: the split hand (Eisen & Kuwabara, 2012), the split hand plus (Menon et al., 2013), the split leg in two versions (leg and foot) (Min et al., 2020; Simon et al., 2015; Wang et al., 2019), and most recently described, the split elbow (Khalaf et al., 2019). For the split hand, there is preferential thenar weakness/wasting compared to the hypothenar hand (Eisen & Kuwabara, 2012). The split hand plus also involves selective weakness/wasting of the flexor pollicis longus (Menon et al., 2013). A recently described split elbow is characterized by preferential involvement of the biceps muscle as compared to the triceps muscle (Khalaf et al., 2019; Thakore et al., 2021). In the split leg, there is preferential plantar‐flexion weakness/wasting compared to dorsiflexion (Simon et al., 2015), but in contradistinction, in the split foot, the extensor digitorum brevis (EDB) is preferentially involved compared to the abductor hallucis (AH) (Min et al., 2020; Wang et al., 2019) (Table 1).

**TABLE 1:** Preferential muscle involvement in ALS split phenotypes and their innervation (gray shading). The anterior horn cells of these muscles receive greatest corticomotoneuronal drive. Green shading denotes muscles with relative sparing, having weaker corticomotoneuronal drive

**Split hand**		
First dorsal interosseus (FDI)	Ulnar	C8, T1
Abductor pollicis brevis (APB)	Median	C8, T1
Abductor digiti minimi (ADM)	Ulnar	C8, T1
**Split elbow**		
Biceps brachii (BB)	Musculocutaneous	C5, C6
Triceps brachii (TB)	Radial	C6, C7, C8
**Split leg/foot**		
Tibialis anterior (TA)	Deep peroneal	L4, L5
Extensor digitorum brevis (EDB)	Deep peroneal	L5, S1
Abductor hallucis (AH)	Tibial/Medial plantar	L5, S1, S2

**FIGURE 1:**
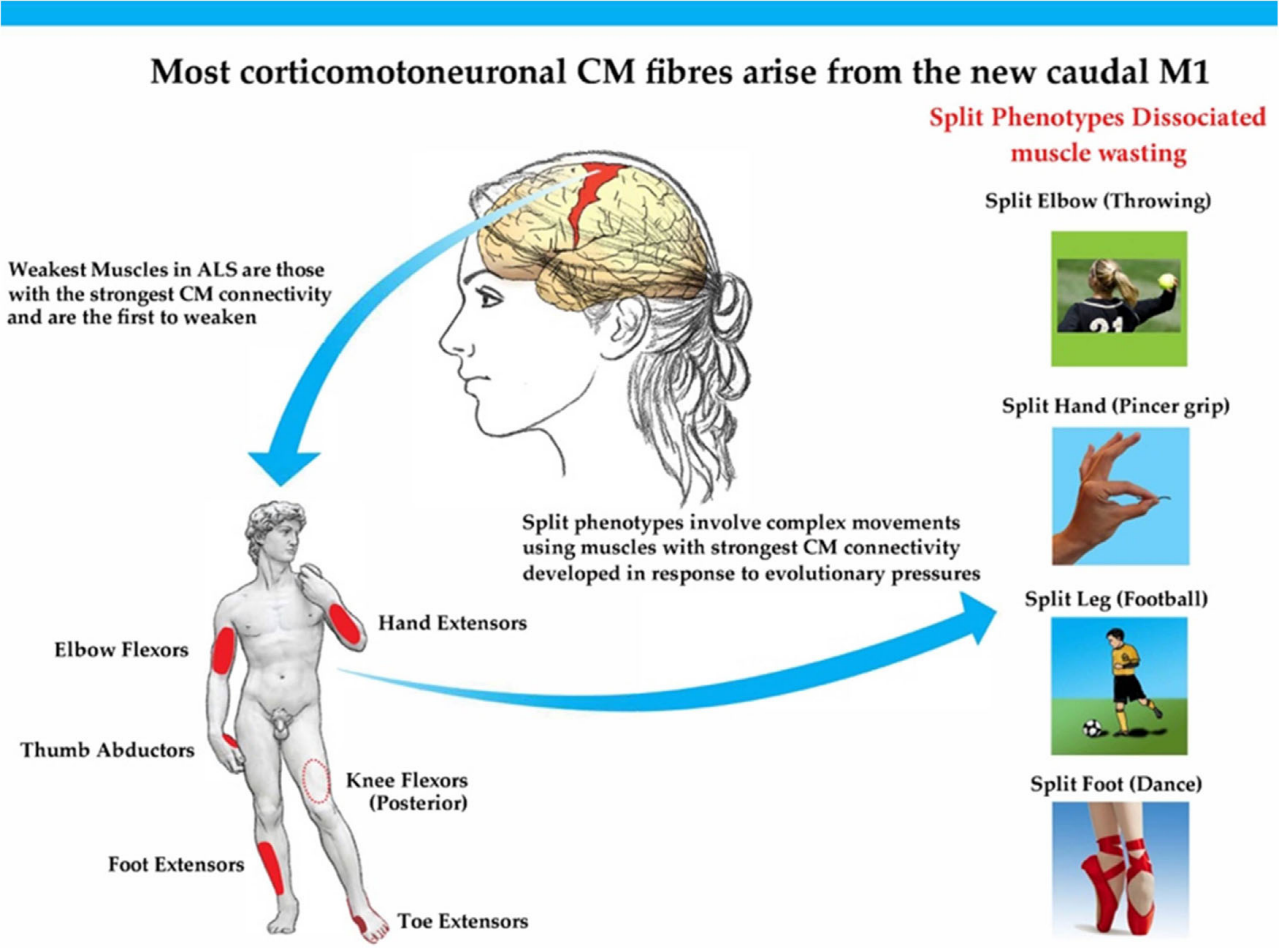
Muscles involved in ALS split phenotypes are those with the strongest CM connectivity used in complex movements developed because of evolutionary pressures

Ludolph et al. (Ludolph et al., 2020), in a large cohort of ALS patients, assessed MRC strength in upper and lower limb muscle pairs, in which one of the pair is known to receive a stronger corticomotoneuronal (CM) drive. The results showed a characteristic pattern of paresis with the muscle having the stronger CM input, that is with more monosynaptic connections, being weaker (lower MRC score). Thus, 1. thumb abductors were weaker than elbow extensors, 2. hand extensors were weaker than hand flexors, 3. elbow flexors were weaker than elbow extensors, 4. knee flexors were relatively weaker than extensors, and 5. plantar extensors were weaker than plantar flexors. Preliminary data also indicate that for the upper limb it is the muscles with stronger CM connectivity that become weak before other muscles, independent of onset site (Thakore et al., 2021) (see Figure 1).

A general concept on the function of the CM system, which is a recently conserved system, is that it subserves complex adaptive motor behaviors which characterize the human motor repertoire (Lemon, 2021). These include skilled tool use, advanced forms of locomotion, for example, required in skiing and football playing, but also when navigating an uneven terrain. Also, CM connections are essential for a wide range of vocalization skills, allowing great variability of tone as used by opera singers (Eisen et al., 2014; Eisen et al., 2017). It is these same highly evolved behaviors that are affected in early ALS, and they are subserved by muscles with the greatest CM connectivity.

The primary motor cortex M1 is subdivided into a caudal region (“new M1”), present only in some higher primates and humans, and a rostral area (“old M1”). The density of cortical spinal tract (CST) neurons is equal in the new and old M1s, but the new M1 contains nearly all of the CM cells making monosynaptic connections with spinal anterior horn cells (Rathelot & Strick, 2009). In ALS, defective motor paradigms are largely subserved by the motor units and their respective muscles having the strongest CM drive, as alluded to the above (Ludolph et al., 2020). We postulate that this is also likely true of the involved (weaker) muscle in the dissociated pair of split phenotypes of ALS, but this remains to be proven. With this in mind, we discuss possible methodologies to investigate ALS split phenotypes specifically from an upper motor neuron perspective, with the intention that this can then be used as early clinical upper motor neuron marker of ALS. Since, several studies have proposed that 1. split phenotypes are not specific to ALS and 2. that they are primarily, or additionally based upon peripheral nerve/muscle dysfunction, we also briefly consider these contradictions to failed CM drive as the primary cause of ALS split phenotypes.

## 2 CORTICOMOTONEURONAL PROJECTIONS

Direct spinal cord connections through CM cells are not present at birth and develop during infancy, when children cultivate an expanding repertoire of movement skills (Armand et al., 1997). These corticomotoneuronal connections are characteristic of all primates (Porter, 1987). Adult humans have monosynaptic CM projections onto all spinal cord motoneurons except those innervating the ocular and sphincter muscles, which are typically spared in ALS until the disease is advanced. Non‐human primates also have significant CM projections to forearm and hand muscles, but outside of these, they are rather limited (Lemon & Griffiths, 2005). Other mammals have indirect, polysynaptic, corticospinal projections, and they do not have a recognizable monosynaptic CM projection, or at least nothing comparable to that of primates (Porter, 1993). This is one important explanation for ALS being uniquely restricted to humans. Indeed, there are no naturally occurring animal models of ALS, and induced ALS animal models, largely studied in rodents, although giving useful information regarding cell function and death, do not truly mimic the human disease (Eisen, 2021).

TAR DNA‐binding protein 43 (TDP‐43) pathology, seen in >95% of patients with ALS, is largely restricted to corticofugal projecting neurons (“dying forward”) (Eisen et al., 2017). Furthermore, the histological patterns of TDP‐43 pathology in the motor cortex are shared in ALS and FTD, whether they occur together or independently. Both Betz cells, other pyramidal corticofugal neurons in the motor neocortex, and alpha‐motoneurons of the lower brainstem and spinal cord become involved at the beginning of the pathological cascade underlying ALS. However, whereas alpha‐motoneurons lose normal nuclear TDP‐43 expression followed by the formation of phosphorylated TDP‐43 aggregates within their cytoplasm, in Betz cells (and other pyramidal corticofugal neurons), TDP‐43 expression is largely unassociated with the development of cytoplasmic aggregations, which remains soluble (Braak et al., 2017). Soluble cytoplasmic TDP‐43 is probably toxic and could enter the axoplasm of Betz cells and other pyramidal neurons, with transmission by axonal transport to the corresponding alpha motoneurons in the lower brainstem and spinal cord, contributing to dysregulation of normal nuclear protein (Braak et al., 2017).

## 3 EXCITATORY POST‐SYNAPTIC POTENTIAL (EPSP) AS A MEASURE OF CM PROJECTION

**FIGURE 2:**
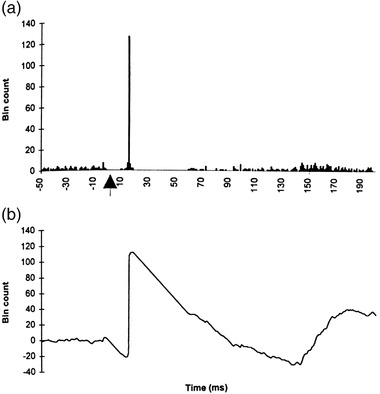
(a) Cumulative sum (CUSUM) analysis of the effect of transcranial magnetic stimulation on the firing probability (peristimulus time histogram) of a single first dorsal interosseous motor unit. A total of 200 randomly stimuli were delivered at time 0 ms (arrow). At about 20 ms after the stimulus, there is a very marked increase in the firing of the motor unit, evident by a sharp upward deflection in the CUSUM. The size of the spike reflects the amplitude of the composite excitatory post‐synaptic potential (EPSP). A period of inhibition lasting about 55 ms follows the increased firing of the motor unit as can be appreciated in (b) which is an expanded view (fast sweep) of (a). The amplitude of the EPSP is measured by the formula: *EPSP(mV) = 100/Number of stimuli x mean interspike interval (ms)/100 ×* *10^−1^ (mV)* In this example it measured 3.9 mV

Transcranial magnetic stimulation (TMS) activates large populations of cortical neurons, and short latency postsynaptic potentials (PSPs) generated in the spinal motoneurons of various limb muscles can be derived from peristimulus time histograms (PSTHs) of repetitively discharging motor units (Ashby & Zilm, 1982). The area of a peak of increased firing probability in a PSTH can then be used to estimate the amplitude of the composite excitatory post‐synaptic potential (EPSP), and the duration of the peak is an estimate of its rise time (Ashby & Zilm, 1982) (see Figure 2). PSTH studies have established that there is pronounced differences in the extent of CM influence between muscle groups acting at different upper and lower limb joints. The CM influence is stronger on wrist extensors compared with wrist flexors, while foot dorsiflexors receive stronger effects than plantar flexors (Bawa et al., 2002; Brouwer & Ashby, 1990; Brouwer & Ashby, 1992; Brouwer et al., 1992; Palmer & Ashby, 1992). These findings fit well with the clinical observations described above by Ludolph et al. (Ludolph et al., 2020).

### 3.1 The split hand

Further, CM influence is task dependent. For example, TMS elicits a larger amplitude motor evoked potential (MEP) in the first dorsal interosseus (FDI) during a pincer and power grip than during a simple index finger abduction. The MEP is of even larger amplitude during pincer gripping than during power gripping (Tinazzi et al., 2003). In essence, the split hand that develops in ALS reflects a failure of pincer grip function (Eisen & Kuwabara, 2012). While CM cells extensively contribute to control tasks requiring fine fractionated digit manipulations, such as a precision grip, increasing evidence suggests that the reticulospinal tract might be well suited to contribute to the control of a power grip (Tazoe & Perez, 2017). In ALS, power grip is maintained for some time after there is loss of fine digit manipulations as required for a pincer grip, which might itself be a useful early clinical measure in ALS.

Measurement of EPSPs as elicited by cortical stimulation has not been performed in the split hand of ALS. It would be of value to compare EPSPs in early ALS prior to significant hand wasting, from the thenar hand (FDI) and abductor pollicis brevis (APB), with those of the abductor digiti minimi muscle (ADM). One would anticipate that there would be a reversal of EPSP size which is normally larger in the FDI and APB and smaller in the ADM.

### 3.2 The split elbow

Corticomotoneuronal excitation occurs mono‐synaptically to upper arm biceps and triceps brachii, but the connections are much stronger to the biceps brachii. The triceps receives a larger portion of polysynaptic (non‐corticomotoneuronal) connections (Brouwer & Ashby, 1990; Palmer & Ashby, 1992). This difference readily explains the split elbow, in which clinically there is preferential weakness in the biceps as compared to the triceps (Khalaf et al., 2019; Vucic, 2019). It is outside the scope of this paper to explore the evolutionary advantages of why some muscles have greater CM connectivity than others, but this is an important question, having relevance to preferential weakness in ALS and split phenotypes. Elbow flexion is vital for virtually all daily activities, particularly reaching and capturing. Also throwing which, maybe, is unique to humans (Lomardo & Deaner., 2918). There is compelling evidence that spoken language shares premotor and motor cortical systems which are also involved in the control of arm gestures, of which elbow flexion is key (Gentilucci et al., 2008). A system sharing hand/arm and mouth function evolved initially in the context of ingestion. Later it formed a platform for combined manual and vocal communication, first as a proto language. In humans, manual gestures are deeply integrated with speech production, and this has relevance to the overlap with ALS‐frontotemporal dementia and the cortical networks they share (Eisen et al., 2014).

### 3.3 The split leg and foot

There is a larger corticomotoneuronal drive projecting to the tibialis anterior (TA) and extensor digitorum brevis (EDB) motoneurons compared with the soleus and the gastrocnemius motoneurons (Advani & Ashby, 1990; Brouwer & Ashby, 1992; Brouwer & Qiao, 1995; Brouwer et al., 1992). This supports the recent observation of Ludolph et al. (Ludolph et al., 2020) that those muscles with stronger corticomotoneuronal influence, dorsiflexors of the foot, are preferentially affected in ALS. Further, an fMRI study has shown that during ankle dorsiflexion, as compared with plantar flexion, significantly more of the contralateral M1 and supplementary motor area (SMA) is recruited (Trinastic et al., 2010). A partial foot drop with tripping, especially associated with more complex movements, such as walking on rough or uneven terrain or skiing, is a common early clinical feature of ALS. Such complex activity is highly dependent on corticomotoneuronal input.

Bipedal walking requires evolutionary musculoskeletal adaptations and accompanying neural computations to manage human locomotion (Capaday, 2002; Nielsen, 2003). The spinal motoneurons for the ankle dorsiflexors, like the wrist extensors, have much stronger CM connections than found in motoneurons subserving ankle plantar flexion (Bawa et al., 2002). When walking on a treadmill, studies in normal subjects using TMS show greater activation of CM input to the tibialis anterior muscle compared to the gastrocnemius (Petersen et al., 2001).

Wang et al. (2019) in describing the split leg in ALS noted preferential impairment of the EDB compared with the abductor hallucis (AH). This was confirmed by Min et al. (2020) who also showed that in ALS, the TA and EDB are more severely affected than the AH. Both these studies were limited to investigating lower motor neuron physiology and lacked any direct assessment of the upper motor neuron. Their observations contradict the earlier conclusions reached by Simon et al. (Simon et al., 2015), the first to describe the “split foot”, who concluded that the planter flexor muscles were preferentially affected in ALS. Again, this study did not directly measure cortical input into the studied muscles, and this makes it difficult to reconcile the different conclusions.

## 4 CORTICOMUSCULAR COHERENCE

Measuring EPSPs using peristimulus time histograms, which is the most direct measure of the size of CM input to a lower motor neuron, is possible but problematical in the lower limbs of ALS patients, and has not been done in this patient population. Using peripheral nerve stimulation and TMS to compare the ratio of the amplitudes of the compound muscle action potential (CMAP) with that of the MEP, as has been done in the split hand, is relatively simple (Weber et al., 2000). Such studies have not been reported in the split elbow, split foot, or split leg of ALS.

A less invasive approach might be to use corticomuscular coherence, an index utilized to indicate coherence between brain motor cortex and associated body muscles (Liu et al., 2019; Liu et al., 2019).

This neurophysiological approach can be applied to examine the integrated physiology of cortical, corticospinal, and neuromuscular systems, and provides a measure of coupling between cortical oscillations and motor unit firing patterns along the corticospinal tract (Mima & Hallett, 1999; Mima et al., 2001). The presence of coherence can be explained by an adequate number of motor neurons receiving temporally synchronized transmissions of synaptic input from cortical projections, coupled with afferent feedback from muscle to spinal and cortical networks (Conway et al., 1995). In ALS, there is a decrease in both corticomuscular and interhemispheric communication during bilateral hand grip. It has been suggested that this is a potential biomarker of motor system dysfunction in ALS, against which to measure future therapeutic efficacy (Proudfoot et al., 2018; Proudfoot et al., 2017).

Corticomuscular coherence has provided evidence of cortical activity associated with normal walking, demonstrating significant causal unidirectional drive from the contralateral motor cortex to muscles during walking (Artoni et al., 2017; Petersen et al., 2012). These findings indicate that the human cortex has a significant role in controlling stereotyped locomotion. Because the cortical topography of muscles is intermixed, cortical localization using corticomuscular coherence may cause difficulty in analyzing split phenotypes, especially in the leg. However, it could be employed to different muscles at the same level of activation to determine whether the coherence is different. Another approach might be to add musculo‐muscular coherence to look for shared drive, comparing muscle pairs in split phenotypes on the most affected side to muscle pairs on the opposite side. Normally, there is not much musculo‐muscular coherence, but particularly on the weaker side of the split phenotype, there might well be an increase in common drive.

## 5 POTENTIAL FOR NEUROIMAGING IN SPLIT PHENOTYPES

Neuroimaging has been extensively utilized in ALS to characterize motor cortex atrophy, corticospinal tract, and brainstem degeneration (Bede et al., 2019; Bede et al., 2016; Querin et al., 2018). Resting‐state fMRI protocols have also been extensively utilized. These data are relatively easy to acquire without the challenges associated with task‐based paradigms (Abidi et al., 2020; Proudfoot et al., 2018). The most commonly used approaches to interpret resting‐state data include graph theory‐based methods (Li et al., 2018; Zhou et al., 2016), independent component analyses (Agosta et al., 2013; Mohammadi et al., 2009; Welsh et al., 2013), and amplitude of low frequency fluctuation methods (Luo et al., 2012; Sako et al., 2017). While decreased sensorimotor (Chenji et al., 2016; Sako et al., 2017; Zhang et al., 2017; Zhou et al., 2014) and cortical‐subcortical (Fekete et al., 2013) network integrity is invariably identified, these observations are solely observed at a broader “motor network” level. They are not specific to the execution of limb or muscle‐group movements, precluding their use in the study of split phenotypes. However, recent methodological developments, including ultra‐high resolution imaging protocols, the availability of high field MRI platforms (7Tesla), and development of novel Non‐Gaussian diffusion models, make current imaging methods ideally suited to investigate the cerebral correlates of split phenotypes (Bede et al., 2018; Kwan et al., 2012).

While each MRI method is associated with specific limitations (resolution/acquisition time/biological value/susceptibility to movement), a combination of several pulse‐sequences overcomes the shortcomings of single imaging methods and a panel of imaging markers is best suited to evaluate specific biological hypotheses (Nasseroleslami et al., 2019). The high resolution of structural sequences complements well the functional insights provided by fMRI. fMRI experiments can be easily tailored to characterize the neural activation patterns of muscles exhibiting dissociate wasting to study the cortical origins of split phenotypes.

## 6 TASK‐BASED IMAGING

Existing imaging initiatives in ALS combine functional, structural, and diffusion sequences optimized for speed of acquisition to accommodate for patients with significant disability. ALS‐related fMRI studies show consensus of task‐based studies which execute motor tasks recruiting pre‐ and supplementary motor areas (Konrad et al., 2002), the ipsilateral motor cortex (Mohammadi et al., 2011; Schoenfeld et al., 2005) subcortical (Konrad et al., 2006; Mohammadi et al., 2011; Tessitore et al., 2006), and cerebellar (Konrad et al., 2006) regions. Task‐based fMRI studies in ALS exemplified by hand opening–closing (Poujois et al., 2013), joystick movement (Stanton et al., 2007), or finger flexion (Konrad et al., 2006) commonly use 1,5 Tesla with voxel sizes ranging from 3 × 3 × 7 (63 mm^3^) to 3.75 × 3.75 × 7 (98.43 mm^3^). These movements are too gross and voxel size too large to detect discrete movement applicable, for example, to the split hand. An appropriate task would be a pincer grip (index–thumb opposition), so far only published as abstracts, but showing expanded cortical activation including the supplementary motor cortex (SMA), premotor cortex (PMA), and sensory cortex (SC) (Brooks et al., 2000). “Motor imagery” which has emerged as an efficient strategy, showing similar activation patterns in ALS and controls as actual movement (Abidi et al., 2020; Lulé et al., 2007; Szameitat et al., 2007), could potentially be applied to all split phenotypes. A complimentary modification is “action observation” which results in cortical activity similar to action execution in ALS (Jelsone‐Swain et al., 2015; Li et al., 2015).

## 7 DESCENDING MOTOR TRACTS

**FIGURE 3:**
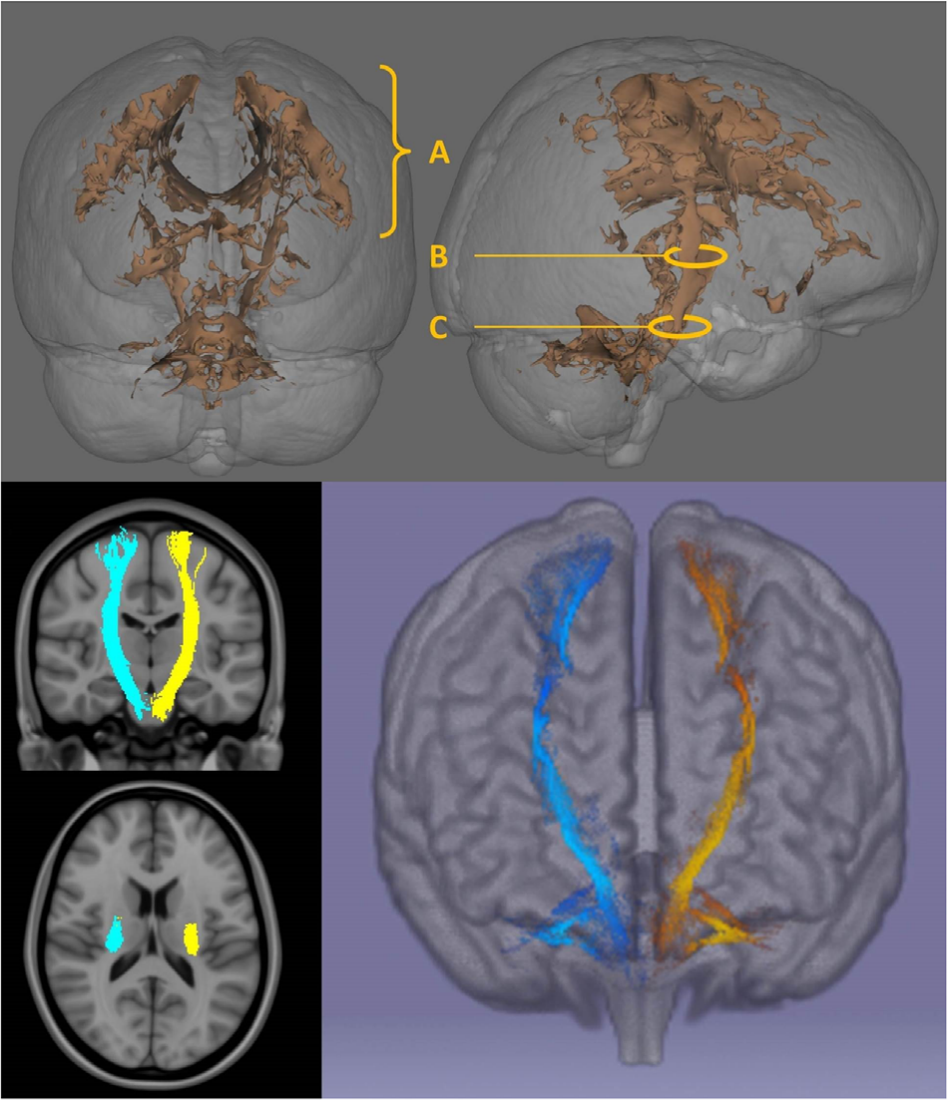
Anatomical patterns of cerebral white matter degeneration in ALS detected by diffusion tensor imaging (DTI). The core white matter signature of ALS includes white matter integrity changes along the entire cerebral course of the corticospinal tracts and degenerative changes in the corpus callosum. Depending on raw data quality and the imaging technique utilized, somatotopic changes can be readily detected subjacent to the primary motor cortex, in the posterior limb of the internal capsule, and to a lesser extent in the mesencephalic cerebral crura. Top: The example shown is a 3D statistical map of fractional anisotropy (FA) changes in 50 patients with ALS compared to 100 healthy controls using tract‐based statistics, permutation‐based testing, and corrections for demographic variables at *p* < .05 FWE TFCE. A: CST changes in the centrum semiovale subjacent to the motor cortex; B: posterior limb of the internal capsule; C: cerebral crus Bottom: CST tractography: coronal views, axial views at the internal capsule, and a 3D reconstruction

Our postulate that split phenotypes are predominately determined by failure of CM drive would be enhanced by anatomical correlates. Corticospinal tract degeneration is readily detected by diffusion tensor imaging in ALS and has been evaluated by a variety of tract‐based and tractographic methods in cross‐sectional and longitudinal analyses. (Bede & Hardiman, 2018) CST integrity is typically characterized by diffusivity metrics (fractional anisotropy, axial‐, radial‐, mean‐diffusivity), and despite sporadic reports (Schuster et al., 2016), segmental changes and somatotopic patterns, which is what is required, are less well characterized (see Figure 3). Nerve fibers in the posterior limb of the internal capsule are somatotopically organized, and this offers an excellent opportunity to evaluate specific body region associated CST degeneration. (Duerden et al., 2011; Pan et al., 2012). Compared to standard diffusion tensor imaging, non‐Gaussian diffusion models, such as neurite orientation dispersion and density imaging have proved particularly sensitive to detect early CST degeneration in both symptomatic (Broad et al., 2019) and presymptomatic cohorts (Wen et al., 2019), and this methodology could be applied to demonstrate early fiber degeneration in split phenotypes.

## 8 THE CONUNDRUM OF PERIPHERAL CAUSATION IN SPLIT PHENOTYPES

Based on evidence outlined above, it is our contention that split phenotypes in ALS are in large part the result of impaired CM drive. Nevertheless, excitability studies have shown altered peripheral motor axonal excitability properties in ALS. Specifically, increases in sodium current (Na^+^) and decreases in potassium (K^+^) current have been found in the motor axons of ALS patients. This in turn may contribute to the development of membrane hyperexcitability in ALS and account for some symptoms such as muscle cramps and fasciculations. For reviews, see references (De Carvalho et al., 2014; Park et al., 2017). Changes in strength–duration time constant and threshold electrotonus have been reported to be more prominent in APB than ADM in the split hand. Based on this and similar studies (Bae et al., 2009; Bae et al., 2013; De Carvalho & Swash, 2019; Shibuya et al., 2013), it has been argued that spinal/peripheral mechanisms also underlie the split hand in ALS, but a subsequent study, using threshold tracking transcranial magnetic stimulation techniques, concluded that cortical hyperexcitability was important to pathophysiology of the split hand (Bae et al., 2014), and in ALS, cortical hyperexcitability precedes lower motor neuron dysfunction (Menon et al., 2015).

In amyotrophic lateral sclerosis (ALS), the large motoneurons that innervate the fast‐contracting muscle fibers (F‐type motoneurons) are vulnerable and degenerate in adulthood. In contrast, the small motoneurons that innervate the slow‐contracting fibers (S‐type motoneurons) are resistant and do not degenerate. Intrinsic hyperexcitability of F‐type motoneurons during early postnatal development has long been hypothesized to contribute to neural degeneration in the adult (Leroy et al., 2014). However, in ALS mutant mice, excitability of F‐type motoneurons is unchanged and S‐type motoneurons of mSDO1 mice did display intrinsic hyperexcitability (lower rheobase, hyperpolarized spiking threshold); so early intrinsic hyperexcitability does not contribute to motoneuron degeneration (Leroy et al., 2014).

We conclude that excitability and similar studies that have suggested that split phenotypes have a spinal/peripheral origin more than likely reflect secondary or compensatory effects, driven by preceding cortical events.

## 9 ARE THERE NON‐ALS SPLIT PHENOTYPES?

As we have stressed, dominant muscle atrophy in the thenar as compared to the hypothenar complex is supportive evidence favoring a primary cortical degeneration in ALS (Eisen, 2021; Lemon, 2021). However, the same phenomenon, both clinically and electrophysiologically, was observed in several other diseases (Kuwabara et al., 2008; Schelhaas et al., 2003), including autosomal dominant spinal muscular atrophy, spinocerebellar ataxia type 3, and juvenile muscular atrophy, leading to the conclusion that the split hand was due to an intrinsic vulnerability of spinal motor neurons subserving the thenar complex (Schelhaas et al., 2003), or even the neuromuscular junction (De Carvalho & Swash, 2019). However, in a large study, Kuwabara (Kuwabara et al., 2008) reported that whereas a decreased APB/ADM ratio, a measure of the split hand, occurred in 41% of ALS patients, it only occurred in 5% of normal controls and 4% of disease controls. Further, the ratio of FDI/ADM was the same in normal and disease controls. There are exceptions to every rule, but when occurring in the right context, the split hand and other split phenotypes are considered ominous, unique to, and diagnostic of ALS.

## 10 CONCLUSIONS

Split phenotypes in ALS are rationally considered to reflect upper motor neuron disease and, in particular, compromise of corticomotoneuronal drive to those motor units innervating the weakened and/or wasted muscles. Further, the distribution of these phenotypes is shared by those muscles which are weakest in ALS, which are muscles also controlled through the strongest corticomotoneuronal drive. Split phenotypes are potentially an early clinical upper motor neuron marker of ALS, but this requires upper motor neuron methodological proof. To this end, we have outlined several methodologies which are potentially helpful in documenting a basis of upper motor neuron dysfunction. Recognizing that these are of variable complexity, they are not going to be routinely used, but we envision that if applied to a spectrum of well selected cases to prove the concept, this would be sufficient for split phenotypes to be used as an upper motor neuron measure in the clinic. Why some muscles preferentially receive stronger CM connectivity than others is presently unanswered, but likely relate to evolutionary pressures subserved through the function of these muscles. Pincer grip (split hand) and language gesture and throwing (split elbow) are two such examples. Underlying the split leg and foot are complex activities such as professional football, ballet, and skating. Complex motor skills develop late both phylogenetically and ontogenetically and are underpinned by ample corticomotoneuronal connections. These networks are particularly vulnerable in ALS, which may lead to preferential muscle involvement and manifest in split‐phenotypes.

## FINANCIAL/GRANT SUPPORT

Andrew Eisen did not have any financial/grant support for this work. Peter Bede's contribution is supported by the Spastic Paraplegia Foundation, Inc. (SPF).

## AUTHOR CONTRIBUTION

Both authors (Andrew Eisen and Peter Bede) shared equally in developing the concept of this paper and in its writing.

## CONFLICT OF INTEREST

Neither Andrew Eisen nor Peter Bede has a conflict of interest to declare.

### PEER REVIEW

The peer review history for this article is available at https://publons.com/publon/10.1002/brb3.2403
